# Genome Characterization and Phylogenetic Analysis of the First Bovine Rhinitis B Virus Isolate in China

**DOI:** 10.3389/fvets.2021.721284

**Published:** 2021-09-22

**Authors:** Shao-Lun Zhai, Yi-Lun Xie, Qi Zhai, Xiao-Hui Wen, Dian-Hong Lv, Qin-Ling Chen, Gang Wang, Wen-Kang Wei

**Affiliations:** ^1^Scientific Observation and Experiment Station of Veterinary Drugs and Diagnostic Techniques of Guangdong Province, Ministry of Agriculture of Rural Affairs, and Key Laboratory of Animal Disease Prevention of Guangdong Province, Institute of Animal Health, Guangdong Academy of Agricultural Sciences, Guangzhou, China; ^2^Agro-Biological Gene Research Center, Guangdong Academy of Agricultural Sciences, Guangzhou, China

**Keywords:** bovine rhinitis B virus, bovine respiratory disease complex, high-throughput sequencing, complete genome, nucleotide deletion, genetic variation

## Abstract

Bovine rhinitis B virus (BRBV) is an emerging viral species in the genus *Aphthovirus*, family Picornaviridae. Studies suggested that BRBV was considered a potential etiological agent of bovine respiratory disease complex (BRDC). BRBV has been reported in the United States, Sweden, Canada, Japan, and Mexico. However, little information of BRBV was available in China. In this study, we performed viral metagenomic analysis in a calf with respiratory disease. The results showed high abundance (3.85) of BRBV nucleotide and 248 mapped reads in calf samples. Online BLASTn analysis showed that three contigs of those had the highest nucleotide similarity (95%) with one Swedish BRBV isolate (BRBV_SWE1, GenBank accession no. KY432299). To identify the genome characterization of the Chinese BRBV isolate (designated CHN1), six couples of overlapping RT-PCR primers were designed according to genome sequences of BRBV_SWE1. Through gene cloning and splicing, we obtained the genome information of CHN1, possessing 7,465 nucleotides (46.6% G+C). Although CHN1 had the highest nucleotide similarity (95.1%) with BRBV_SWE1, one 11-nucleotide (ACATTTGTTGT) deletion occurred in the 5′ untranslated region compared to SWE1. Phylogenetic analysis showed that CHN1 clustered together with BRBV_SWE1, and far from other BRBV isolates. This study recorded the first discovery of BRBV infection in China. Further investigation should be made in order to evaluate the infection status and epidemiological significance of BRBV in China.

## Introduction

Bovine respiratory disease (BRD), an important multifactorial disease involving complex interactions between the animal, the pathogens, and the environment, severely affected the world bovine industry economically. In the Unites States alone, the economic impact due to BRD is estimated to exceed 1 billion dollars per year (1, 2). The BRD complex accounts for ~70–80% of morbidity in the Unites States and 84.5–99.9% of morbidity in Mexican feedlot cattle (3, 4). In Canada, over 80% of the vaccines licensed for cattle are applied for control and prevention of BRD (5, 6). BRD poses significant challenges to its prevention and control (7).

To date, major pathogens responsible for BRD include *Histophilus somni, Pasteurella multocida, Mannheimia haemolytica*, bovine herpesvirus 1 (BHV-1), bovine viral diarrhea virus (BVDV), bovine parainfluenza 3 virus (PI3V), and bovine respiratory syncytial virus (BRSV) (8). Despite the aggressive use of antibiotics and vaccines against the above pathogens, the morbidity and mortality rates of BRD among feedlot cattle have remained steady (3). This may be due to the etiological role of some new pathogens including bovine adenovirus 3 (BAdV3), influenza D virus (IDV), and bovine rhinitis virus (BRV) (9).

BRV, along with equine rhinitis virus (ERV) and foot-and-mouth disease virus (FMDV), is a species in the genus *Aphthovirus*, family Picornaviridae, according to the International Committee on Taxonomy of Viruses. BRV has two species, bovine rhinitis A virus (BRAV) and bovine rhinitis B virus (BRBV) (10). BRBV has been reported in bovine herds from the United States, United Kingdom, Mexico, Sweden, and Canada, and was considered to be associated with BRD (9, 11–14). However, little information of BRBV was available in China (15). In this study, we performed viral metagenomic analysis in a calf with respiratory disease. A nearly complete genome sequence of the first Chinese BRBV isolates was obtained and characterized.

## Materials and Methods

### Sample Information

On March 11, 2014, one dead Holstein calf was submitted to the Institute of Animal Health, Guangdong Academy of Agricultural Sciences. According to the calf owner, the calf before death mainly presented dyspnea and diarrhea. Autopsy result was consistent with the description of the calf owner. The pathological changes mainly focused on lung (pneumonia) and intestines (increased watery contents), and parasitic agents were not observed. To further identify the possible pathogens causing the death of the calf, the spleen and blood were collected to culture the bacteria, and the lung and intestine tissues were collected to perform viral metagenomic analysis.

### Viral Metagenomic Analysis

Prior to viral RNA/DNA extraction, the lung and intestine tissues were ground and diluted one time using phosphate-buffered saline (PBS) (pH: 7.4). The supernatants were then collected by centrifugation at 5,000 × *g* for 5 min. Two hundred microliters of clarified supernatants was used to extract nucleotide following the manufacturer's recommendations (Axygen Scientific Inc., Union City, CA, USA). The nucleotide samples (100 μl) were mixed together and submitted to a sequencing company (Annoroad Gene Technology Inc., Beijing, China) to perform high-throughput sequencing. The results showed high abundance (3.85) of BRBV nucleotide and 248 mapped reads in the calf samples.

### Genome Amplification of BRBV

From the three fragments of spliced sequences (446, 465, and 482 bp), the online BLASTn analysis revealed that they shared the highest nucleotide similarity (95%) with one Swedish BRBV isolate (GenBank no. KY432299). So, according to the genome sequence of the Swedish BRBV isolate, six overlapping pairs of primers (Table 1) were designed and used to amplify the genome sequence of the Chinese BRBV isolate. The 25-μl one-step RT-PCR reaction mixture included 12.5 μl of 2 × 1 Step Buffer, 1 μl of PrimeScript one Step Enzyme Mix, 1 μl of upstream primer (10 μM), 1 μl of downstream primer (10 μM), 2 μl of RNA sample, and 7.5 μl of RNase-Free ddH_2_O. RT-PCR protocol was designed as follows: reverse transcription at 50°C for 30 min, pre-denaturation at 95°C for 5 min, 45 cycles of (denaturation at 95°C for 30 s, annealing at optimized temperature listed in Table 1 for 30 s, extension at 72°C for 2 min), and final extension at 72°C for 5 min. The positive amplicons were cloned into the pMD19-T vector (Takara, Shiga, Japan), and all positive recombinant plasmids were submitted to a sequencing company (Sangon Biotech Co., Ltd., Shanghai, China) and sequenced for at least three times. One nearly complete genome sequence of BRBV (designated CHN1) was obtained (Table 2), and has been submitted to the GenBank database (accession number MT160419).

**Table 1:** RT-PCR primers used in this study.

**Fragment**	**Primers**	**Length (bp)**	**Annealing temperature (^**°**^C)**
1	1-F: 5′-CGGGTTTGCTGCTTTCACAT-3′	2,169	58
	2,169-R: 5′-CCTTCGTAGCAACGGGCAAA-3′		
2	1,698-F: 5′-GGGCACCGTTGGACTATGAA-3′	1,737	60
	3,434-R: 5′-TTGTATGAAACGTGGGCAGTA-3′		
3	3,275-F: 5′-CACTTACAACGGCACTACATTCCACG-3′	1,820	60
	5,094-R: 5′-CCTCGTCGCATTCCTCGTTGTA-3′		
4	4,993-F: 5′-TTAACGCCAGTCCTGAGATGC-3′	722	57
	5,714-R: 5′-AACAACTGGTGAGTTCCTGGTAA-3′		
5	5,458-F: 5′-TCGCTACTTGCTGTGCTCTTGG-3′	1,789	56
	7,246-R: 5′-CGCCGTGCGAAGGACAGGAT-3′		
6	6,846-F: 5′-GCTTATGAGGACAAGCGTATCAC-3′	620	56
	7,465-R: 5′-TGTGCCGAATTGTCCCAAAT-3′		

*The position of primers was from CHN1 (GenBank accession number: MT160419)*.

**Table 2:** Information of members in the genus *Aphthovirus* used in this study.

**No**.	**Species**	**Isolate**	**Genome** **(nt)**	**Polyprotein** **gene (nt)**	**Country**	**Collection** **time**	**GenBank** **number**
1	BRAV	140032-1	7,235	6,732	USA	2013	KP236129
2	BRAV	H-1	7,250	6,657	Japan	1984	JN936206
3	BRAV	Sd-1	7,245	6,657	Germany	1962	KP236128
4	BRAV	BSRI4	7,207	6,681	USA	2013	KP264974
5	BRAV	RS3X	7,267	6,642	UK	1969	KT948520
6	BRBV	BRBV_SWE1	7,476	6,825	Sweden	2014	KY432299
7	BRBV	TCH5	7,474	6,825	USA	2013	KU168861
8	BRBV	MexB09	7,494	6,852	Mexico	2015	KU159360
9	BRBV	EC11	7,556	6,843	UK	Unavailable	EU236594
10	BRBV	140032-2	7,499	6,843	USA	2013	KP236130
11	BRBV	BSRI1	7,359	6,843	USA	2013	KP264980
12	BRBV	MexB48	7,414	6,843	Mexico	2015	KU159361
13	BRBV	TCH8	7,484	6,843	USA	2013	KU168862
14	BRBV	CHN1	7,465	6,825	China	2014	MT160419
15	FMDV	A10 Holland	8,161	6,999	Netherlands	1942	AY593751
16	FMDV	Akesu/58	8,147	6,999	China	1958	AF511039
17	FMDV	YNBS/58	8,163	6,990	China	1958	AY390432
18	FMDV	C3 Indaial	8,183	6,987	Brazil	1971	AY593806
19	FMDV	SAT1-1bech	8,173	7,020	Botswana	1970	AY593838
20	FMDV	SAT2-2 106/67	8,135	7,008	Unavailable	1967	AY593848
21	FMDV	SAT3-3kenya 11/60	8,164	7,008	Kenya	1960	AY593852
22	ERAV	PERV-1	7,782	6,747	USA	Unavailable	DQ272578
23	ERAV	D1305-03	7,681	6,747	UAE	2003	KM269483
24	ERAV	PERV	7,734	6,684	Unavailable	Unavailable	X96870

### Sequence Analysis

Sequence alignment analysis based on CHN1 and reference BRAV, BRBV, FMDV, and ERV sequences (Table 2) was performed using the Clustal W program implemented in the Lasergene v7.1 sequence analysis software package (DNASTAR Inc., Madison, WI, USA). A phylogenetic tree was then constructed based on the polyprotein gene of three of viral species in the genus *Aphthovirus* by the Maximum Likelihood method using the Molecular Evolutionary Genetics Analysis (MEGA) software version 7.0 with bootstrap replication at 1,000 (16). Similarity plot for CHN1 against EC11, TCH5, and SWE1 was generated using the Kimura (2-parameter) distance model with a 200-bp window and the 20-bp step in the software SimPlot v3.5.1 (17).

### Isolation of BRBV-CHN1

The supernatant of the homogenate of the lungs and intestine tissues from the calf in this study was filtered with a 0.22-μm filter, inoculated to a monolayer of Madin-Darby bovine kidney (MDBK) cells, and incubated at 33°C with concentration of CO_2_ at 5% for 120 h. The supernatant of the culture went on three passages. Each passage was observed for any CPE, and the supernatants of every passage were extracted (Axygen Scientific Inc.). RT-qPCR was run on the extracts to detect any viral replications.

## Results and Discussion

Through RT-PCR amplification, a nearly full-length genome sequence (7,465 nucleotides, 46.6% G+C) of the first Chinese BRBV isolate (CHN1) was obtained, which shared the highest nucleotide similarity (95.1%) with BRBV_SWE1. Overall, CHN1 shared 77.9–95.1% similarity with the selected BRBV isolates, 55.5–56.2% with FMDV, 53.4–54.5% with BRAV, and 8.2–27.6% with ERAV on the level of genomics (Table 3).

**Table 3:** Similarity of CHN1 with other member from the genus *Aphthovirus*.

**Species**	**Isolate**	**Complete genome** **(nucleotide)**	**Polyprotein gene** **(nucleotide)**	**Polyprotein** **(amino acid)**
BRAV	140032-1	53.4%	57.4%	50.2%
BRAV	H-1	53.6%	58.4%	50.8%
BRAV	Sd-1	53.9%	58.7%	51.2%
BRAV	BSRI4	54.5%	58.8%	50.9%
BRAV	RS3X	53.5%	58.4%	51.2%
BRBV	BRBV_SWE1	95.1%	94.9%	98.5%
BRBV	TCH5	80.1%	79.4%	88.3%
BRBV	MexB09	78.2%	78.2%	85.9%
BRBV	EC11	79.0%	78.5%	85.9%
BRBV	140032-2	78.8%	78.4%	85.8%
BRBV	BSRI1	77.9%	78.2%	86.0%
BRBV	MexB48	78.2%	78.2%	85.8%
BRBV	TCH8	78.1%	78.3%	86.0%
FMDV	A10 Holland	56.1%	56.6%	46.5%
FMDV	Akesu/58	55.5%	56.9%	45.8%
FMDV	YNBS/58	55.5%	57.1%	46.4%
FMDV	C3 Indaial	55.8%	57.4%	46.4%
FMDV	SAT1-1bech	55.7%	56.0%	46.3%
FMDV	SAT2-2 106/67	55.9%	57.0%	45.8%
FMDV	SAT3-3kenya 11/60	56.2%	57.4%	45.8%
ERAV	PERV-1	8.2%	9.1%	38.3%
ERAV	D1305-03	27.6%	7.3%	27.6%
ERAV	PERV	8.2%	3.5%	27.6%

It is noteworthy that CHN1 shared only 79.0% similarity on the level of complete genome and 85.9% on the level of polyprotein with the BRBV isolate EC11 that was first identified in England, for the reason that EC11 is the only isolate studied well on the level of genomics so far. Thus, the genomic regions of CHN1 genome in this study were mainly predicted based on the genomic characteristics of EC11 (18).

**Figure 1:**
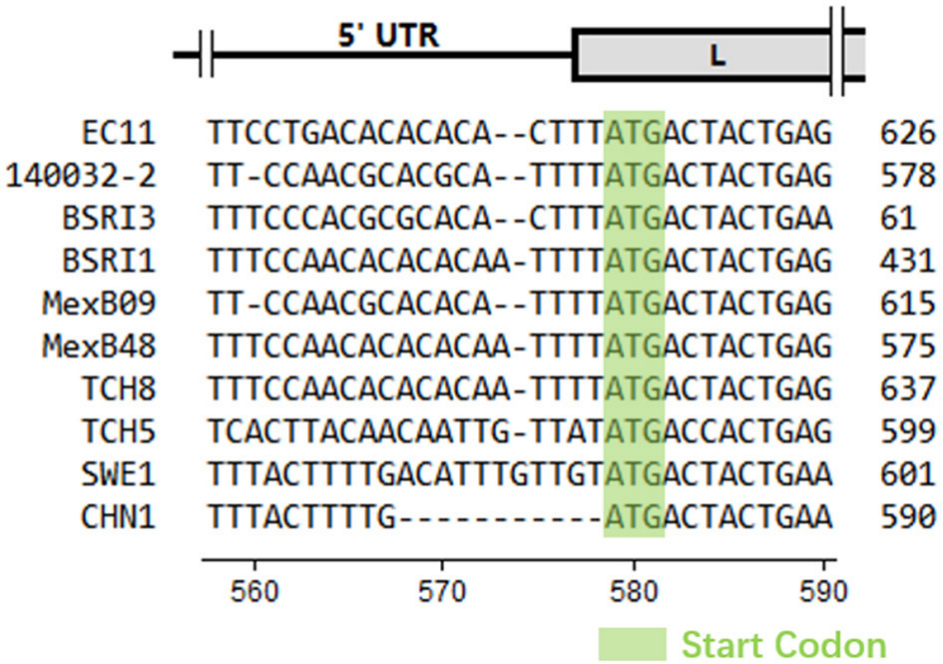
Partial nucleotide alignment of CHN1 with reference sequences. The absence of nucleotides was represented by a dot. The scale of genomic regions was illustrated according to EC11 predicted in a previous study (18) and the position scale was indicated for CHN1. Start codons were indicated with a light green background.

CHN1 had the closest relationship with SWE1. In the middle of CHN1 genome, there was a large complete open reading frame (ORF) (6,825 nt) (94.9% with SWE1) that encodes the polyprotein (2274 amino acid) (98.5% with SWE1). Meanwhile, the nearly complete 5′ and 3′ untranslated regions of CHN1 genome that had been successfully sequenced were 578 nucleotides (94.9% with SWE1) and 62 nucleotides (100% with SWE1) in length, respectively. However, compared with SWE1, one 11-nucleotide (ACATTTGTTGT) deletion occurred before the Leader region compared to SWE1 (Figure 1).

**Figure 2:**
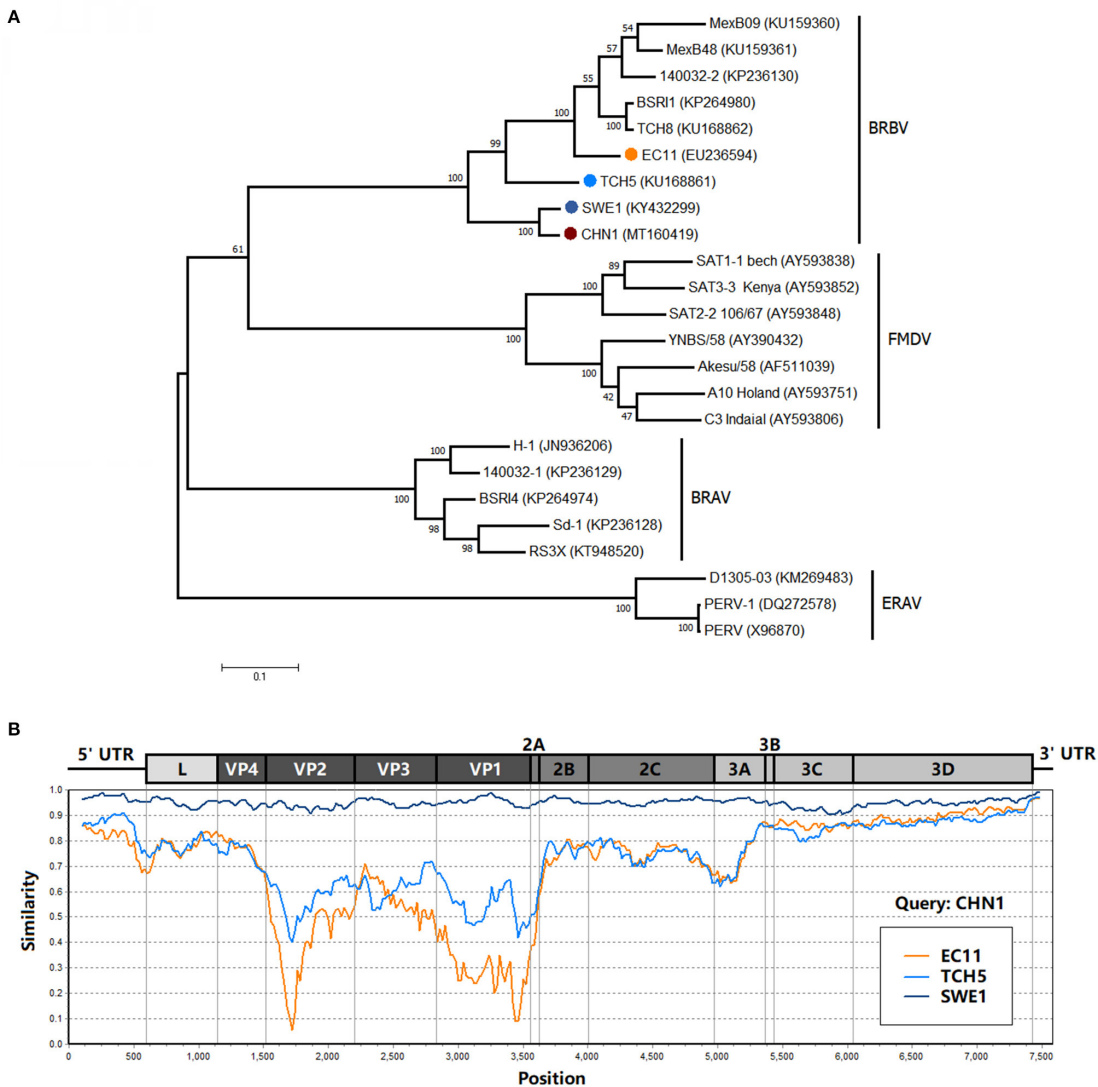
**(A)** Phylogenetic analysis of BRBV polyprotein sequence with other viruses from the genus *Aphthovirus*. The phylogenetic tree was drawn to scale using the Maximum Likelihood method and tested with the booststrap method at a replication of 1,000, and its frequency was marked at the nodes. The isolate CHN1 that was identified in this study was marked in bold and accompanied by a red circle. **(B)** Similarity analysis of isolate CHN1 with isolates EC11, TCH5, and SWE1 using the Kimura (2-parameter) distance model with a 200-bp window and 20-bp step. The genomic region scale was illustrated according to isolate EC11.

Phylogenetic analysis of the polyprotein genes showed that CHN1 clustered together with BRBV_SWE1, and was closer to the BRBV isolate TCH5 identified in United States but far from other BRBV isolates (including EC11) and related viral species (Figure 2A). Further investigation on the similarity between EC11, SWE1, TCH5, and CHN1 complete genome (Figure 2B) revealed that the 5′ NTR, L, VP4, 2BC, 3ABCD and 3′ NTR region of the selected isolates shared >60% of similarity, manifesting relatively high genomic conservation. On the contrary, VP2, VP3, VP1, and 2A region shared similarity ranging from 5 to 75%, which was more diverse compared to the rest of the regions mentioned above. For picornaviruses, generally, the continuous genomic region P1 encodes the structural proteins VP4, VP2, VP3, and VP1 that compose the viral capsid. In FMDV, VP1 is the most exposed protein on the viral capsid surface (19) while VP4 is an internal structural protein that is conversed in different serotypes (20), which accords with the similarity analysis where VP4 shared 65–80% similarity while VP2, VP3, and VP1 shared 5–70% among selected BRBV isolates. In addition, like EC11 and SWE1, the 2A/2B junction of CHN1 preserved the four-residue “NPGP” that involved a “cleavage” mechanism termed “ribosomal skipping” that was demonstrated in FMDV (21).

BRBV could exist worldwide. In a span of a relatively short period of time (2013–2015), with the next-generation sequencing technic thriving, reports of BRBV from different countries increased and most of the cases had connections with BRD. In an early study into the pathology of BRBV, increased body temperature, increased respiration rate, serious nasal discharge, and lesions in the lungs were observed (22). Similar conditions were noticed in the calf in this study and the calves in the studies mentioned above. In particular, the metagenomic analysis from this study confirmed no other common BRD-related virus presented, while the autopsy result showed that the infection in the calf was rather viral, and no bacteria had been isolated *in vitro*, indicating the potential pathogenicity of BRBV. However, although attempts were made to isolate the BRBV-CHN1, neither CPE- nor BRBV-positive RT-qPCR reaction was observed in all three passages. Therefore, further investigation and more evidence are needed to extrapolate the theory.

Generally, BRBV was frequently detected in those respiratory tract samples (such as nasal swabs, lungs) (9, 11, 13, 14). Little information was available in other samples. In this study, the nucleotide sample using viral metagenomic analysis was the mixture from lung and intestine tissues; this does not determine whether the virus comes from the lung or small intestine. However, further detection showed that BRBV was only detected in the lung sample rather than other samples (intestine tissue, spleen, and blood). Moreover, in our previous study, we detected a lower positive number of BRBV in stool samples than in the nasal swab (15). To a certain extent, it suggested that BRBV could spread via the respiratory tract and/or fecal–oral transmission.

## Conclusions

This study recorded the first discovery of BRBV in China. Further investigation should be made in order to evaluate infection status and epidemiological significance of BRBV in China.

## Data Availability Statement

The datasets presented in this study can be found in online repositories. The names of the repository/repositories and accession number(s) can be found in the article/supplementary material.

## Ethics Statement

The animal study was reviewed and approved by Institute of Animal Health, Guangdong Academy of Agricultural Sciences. Written informed consent was obtained from the owners for the participation of their animals in this study.

## Author Contributions

S-LZ: conceptualization. S-LZ and Y-LX: methodology and writing—original draft preparation. Y-LX: software. S-LZ, Y-LX, QZ, X-HW, D-HL, and Q-LC: writing—review and editing. W-KW: supervision. S-LZ, GW, and W-KW: project administration. All authors contributed to the article and approved the submitted version.

## Funding

This work was supported by the Jinying's Star Talent Program (Grant no. R2018PY-JX003) from Guangdong Academy of Agricultural Sciences, the grant (No. 201906040005) from Guangzhou Science and Technology Bureau, the grant (No. 2019B020217002) from the Guangdong Science and Technology Department, and the grant (Nos. 2020KJ114 and 2020KJ119) from the Department of Agriculture and Rural Affairs of Guangdong Province.

## Conflict of Interest

The authors declare that the research was conducted in the absence of any commercial or financial relationships that could be construed as a potential conflict of interest.

## Publisher's Note

All claims expressed in this article are solely those of the authors and do not necessarily represent those of their affiliated organizations, or those of the publisher, the editors and the reviewers. Any product that may be evaluated in this article, or claim that may be made by its manufacturer, is not guaranteed or endorsed by the publisher.
